# Supplementary material for: Effect of One Step Solid State Reaction Route on the Semiconductor Behavior of the Spinel (NI, Co, and Mn)O4 to Be Used as Temperature Sensor
Source: Sensors (Basel). 2023 Jun 6;23(12):5380. doi: 10.3390/s23125380 (PMC10301229; doi:10.3390/s23125380)
Supplement: Supplementary file 1 [file sensors-23-05380-s001.zip › sensors-2426668-supplementary.pdf]

Supporting Information

Effect of One Step Solid State Reaction Route on the Semiconductor Behavior of the Spinel (NI, Co, and Mn)O4 to Be Used as Temperature Sensor

Daehyeon Ko and Sungwook Mhin *

Department of Advanced Materials Engineering, Kyonggi University, Suwon 16227, Korea

* Correspondence: swmhin@kgu.ac.kr

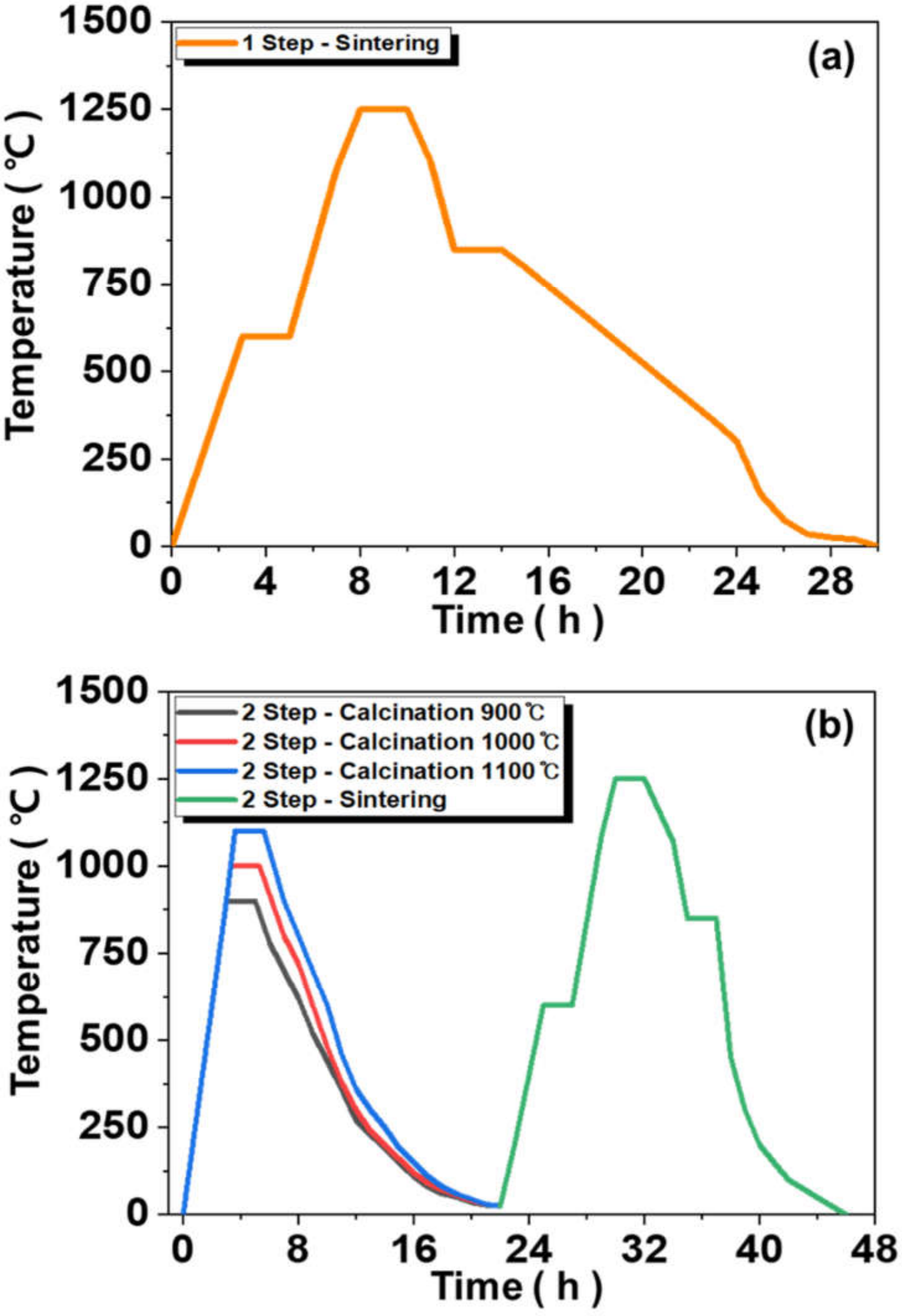

Figure S1. Heating profile of (a) 1-step SSR route and (b) 2-step SSR route.

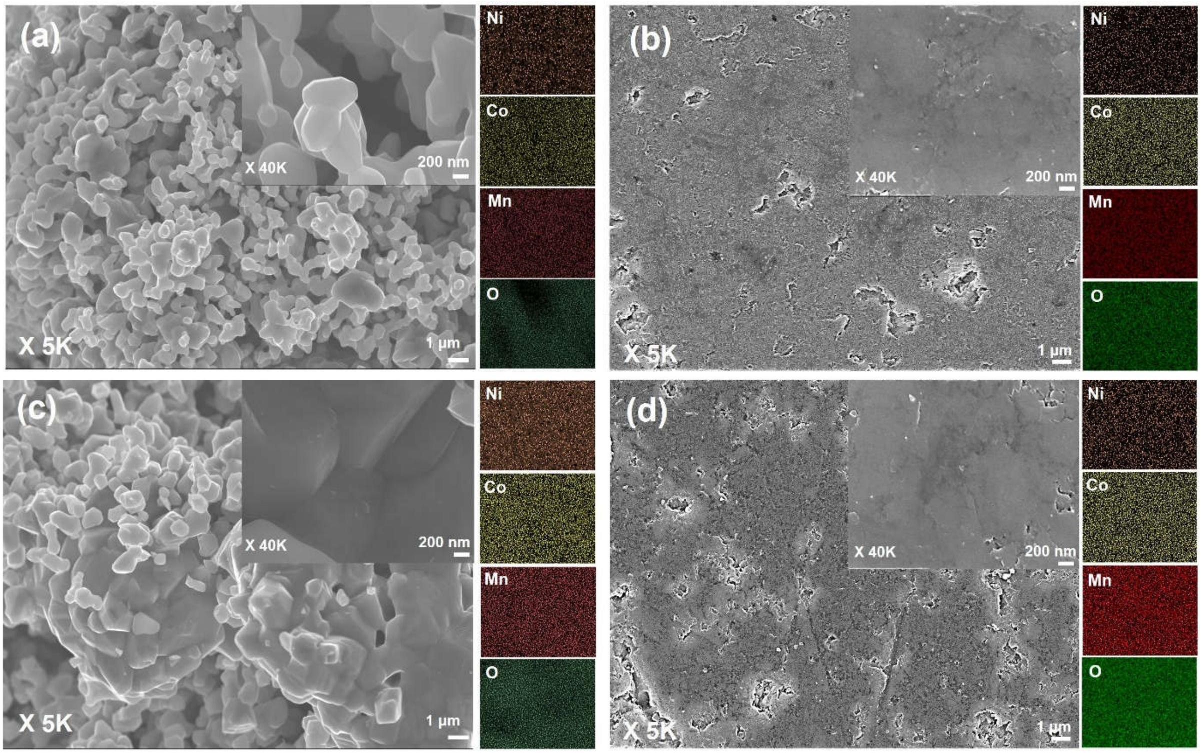

Figure S2. SEM images of the NMC via (a) a 2-step SSR route after calcination (900 °C) and (b) a 2-step SSR route after sintering (900 °C), and via (c) a 2-step SSR route after calcination (1000 °C) and (d) 2-step SSR route after sintering (1000 °C).

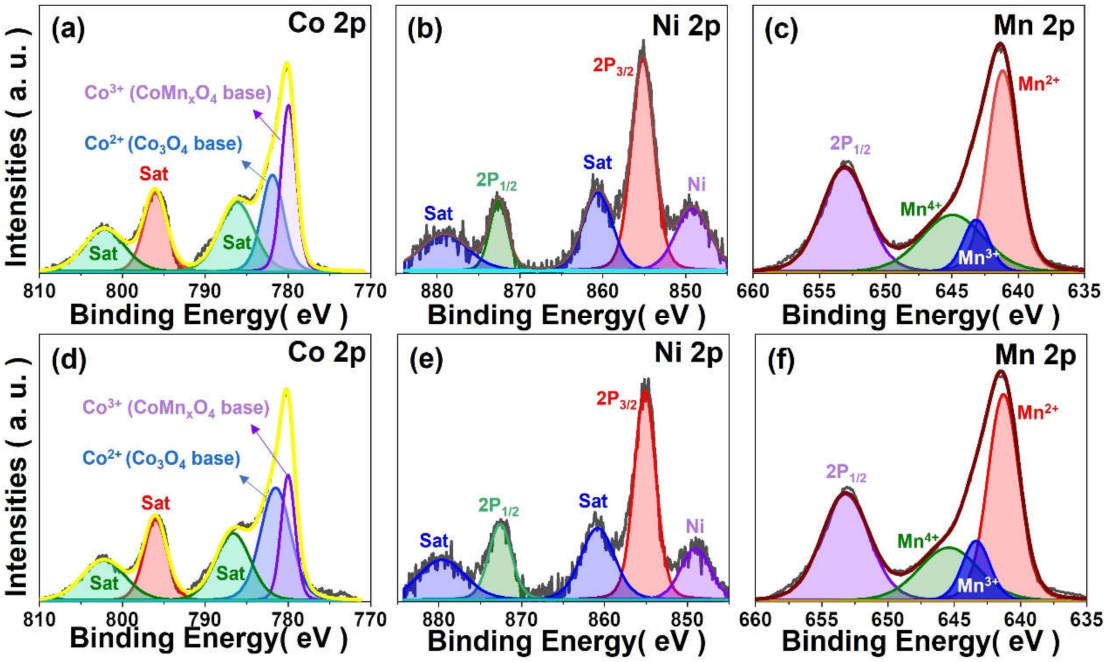

Figure S3. XPS spectra of (a)Co 2p (b)Ni 2p (c)Mn 2p of the NMC via 2-step SSR route (calcination temperature: 900 °C), and (d)Co 2p (e)Ni 2p (c)Mn 2p of the NMC via 2-step SSR route (calcination condition: 1000 °C).

Table S1. The results of XPS elemental analysis for (a) Co 2p, (b) Ni 2p, and (c) Mn 2p from NMC via 2-step SSR route (calcination temperature: 1100 °C), and (d) Co 2p, (e) Ni 2p and (f) Mn 2p from NMC via 1-step SSR route.

**2-Step SSR (1100 °C)**	**(a) Co 2P**		**(b) Ni 2p**	**(c) Mn 2p**		
	**Co^{2+}**	**Co^{3+}**	**Ni^{2+}**	**Mn^{4+}**	**Mn^{3+}**	**Mn^{2+}**
Area(%)	49.89	50.11	100	25.95	26.02	48.03
1-Step SSR	(d) Co 2p		(e) Ni 2p	(f) Mn 2p		
	Co^{2+}	Co^{3+}	Ni^{2+}	Mn^{4+}	Mn^{3+}	Mn^{2+}
Area(%)	51.75	48.25	100	35.22	35.40	29.38

Table S2. The results of XPS elemental analysis for O 2p from NMC via (a) 2-step SSR route (calcination temperature: 1100 °C) and (b) 1-step SSR route.

	**(a) 2-step SSR (1100 °C) O 1S**			**(b) 1-step SSR O 1S**		
	**O_C**	**O_V**	**O_L**	**O_C**	**O_V**	**O_L**
Area(%)	12	50	37	8	55	37

Table S3. B value and resistivity (ρ) at room temperature (25 °C) of the NMC via a 2-step SSR route and 1-step SSR route.

	**B value**	**Resistivity ($\rho_{25\,°C}$)**
2-step Sintering 900 °C	3671	397.83
2-step Sintering 1000 °C	3727	589.41
2-step Sintering 1100 °C	3551	442.35
1-step Sintering	3549	12.05
